# Comparative Roles of Hydrogels, Deep Eutectic Solvents, and Ionic Liquids in Enzyme-Based Biosensors, Bioelectronics and Biomimetics Devices

**DOI:** 10.1021/acsmeasuresciau.5c00036

**Published:** 2025-07-15

**Authors:** Fhysmélia Firmino de Albuquerque, Rodrigo Michelin Iost, Frank Nelson Crespilho

**Affiliations:** a São Carlos Institute of Chemistry, University of São Paulo (USP), São Carlos 13560-970, Brazil; b Department of Fundamental Chemistry, Institute of Chemistry, University of Sao Paulo, Av. Professor Lineu Prestes, 748-B4T, Butantã, Sao Paulo 05508-000, Brazil

**Keywords:** enzyme stabilization, enzyme immobilization, bioinspired systems, hybrid biointerfaces, biocompatibility, soft materials, functional materials, electrochemical sensing

## Abstract

The development of enzyme-based bioelectronic devices, including biosensors and biomimetic systems, has significantly advanced with the introduction of innovative materials such as hydrogels, deep eutectic solvents (DES), and ionic liquids (ILs). These materials offer unique advantages in enhancing biodevice performance, particularly in enzyme stabilization, biocompatibility, and electrochemical sensitivity. Hydrogels, known for their high water content and flexibility, provide an ideal matrix for enzyme immobilization in biological applications but are limited by low ionic conductivity. DES, with their green chemistry credentials and ability to stabilize enzymes under harsh conditions, show great promise, although scalability and performance in complex biological systems remain challenges. ILs, with their superior electron transfer capabilities, enable high sensitivity in electrochemical biosensors, though issues of viscosity and potential toxicity need to be addressed for broader biomedical use. This review provides a comparative analysis of the roles of these materials in enzyme-based biosensors and bioelectronics, including microbatteries and bioelectrosynthesis, highlighting their respective strengths, limitations, and future opportunities. The integration of these materials holds great potential for advancing bioelectronics technologies, with applications spanning medical diagnostics, environmental monitoring, and industrial processes. By addressing current challenges and optimizing these materials for large-scale use, the future of enzyme-based devices could see significant improvements in efficiency, sensitivity, and sustainability.

## Graphical abstract

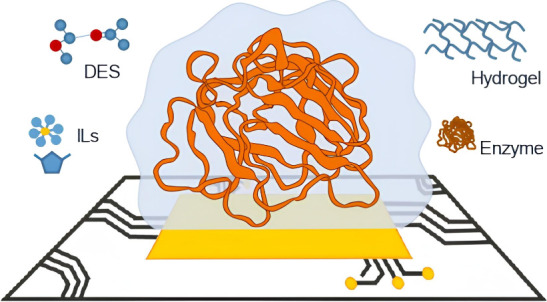

## Introduction

The rapid advancement of biosensors and bioelectronics has been driven by the growing need for more sensitive, accurate, and sustainable devices in healthcare, environmental monitoring, and bioengineering.^1,2^ These devices rely on advanced materials that can seamlessly interface with biological systems while offering high performance and durability.^3^ Additionally, innovative platforms such as flexible carbon fiber electrodes have demonstrated exceptional performance, as seen in their application for ultrasensitive SARS-CoV-2 detection in human saliva,^4^ highlighting the importance of material advancements in modern biosensors. This exemplifies how technological innovation in biosensing has been driven not only by material improvements but also by broader global demands. Recent discussions highlight how the COVID-19 pandemic, for instance, acted as a catalyst for accelerating the integration of research, business, and innovation, reinforcing trends that were already shaping the future of biosensor and bioelectronics development and market competitiveness.^5^

Beyond biosensors, a wide range of bioelectronic devices has emerged, including biofuel cells,^6^ implantable biodevices,^7^ neural interfaces,^8^ wearable sensors,^9^ lab-on-a-chip platforms,^10^ and bioelectronic medicine.^11^ Biofuel cells leverage enzymatic or microbial catalysis to generate electricity from biological substrates, paving the way for self-powered biosensors and medical implants. Neural interfaces, such as brain-computer interfaces, rely on biocompatible materials and signal processing advancements to enable communication between electronic systems and neural networks, offering groundbreaking applications in prosthetics, neuromodulation, and cognitive enhancement. Wearable sensors, utilizing flexible and stretchable materials, are revolutionizing real-time health monitoring, providing continuous physiological data with minimal user discomfort. Lab-on-a-chip platforms integrate microfluidics, biosensing, and electronics to enable rapid, high-throughput diagnostics, which are crucial for disease detection and personalized medicine. Bioelectronic medicine, a rapidly growing field, explores electronic modulation of biological pathways to treat conditions such as inflammation, pain, and neurological disorders.

The development of enzyme-based bioelectronic devices, including biosensors and biomimetic systems, has significantly advanced with the introduction of innovative materials such as hydrogels, deep eutectic solvents (DES), and ionic liquids (ILs).^12^ These materials offer unique advantages in enhancing biosensor performance, particularly in enzyme stabilization, biocompatibility, and electrochemical sensitivity. Hydrogels, known for their high water content and flexibility, provide an ideal matrix for enzyme immobilization in biological applications.^13^ DES, with their green chemistry credentials and ability to stabilize enzymes under harsh conditions, show great promise, although scalability and performance in complex biological systems remain challenges. ILs, with their superior electron transfer capabilities, enable high sensitivity in electrochemical biosensors, though issues of viscosity and potential toxicity need to be addressed for broader biomedical use.

In addition to biomedical and diagnostic applications, bioelectronic devices are playing a critical role in sustainable energy generation and environmental remediation. Enzymatic and microbial biofuel cells offer a promising avenue for clean energy production, efficiently converting organic substrates into electricity. Bioelectrosynthesis, a process leveraging electrogenic microorganisms, enables the sustainable production of valuable chemicals, including biohydrogen and biofuels, with minimal carbon emissions.^14^ Water splitting technologies, inspired by natural photosynthesis, are being explored using biohybrid catalysts to facilitate hydrogen production as a renewable energy source.^15^ Furthermore, bioelectronic systems are being integrated into carbon capture and utilization strategies, employing engineered microbial systems to convert CO_2_ into biobased products. These advancements in bioelectrochemical systems contribute to global efforts toward decarbonization, fostering the development of sustainable energy solutions and circular bioeconomy models.

DES, ILs, and hydrogels have emerged as key materials that can address many of the challenges in current bioelectronic technologies **(** Figure 1 **)**.^16–19^ Deep eutectic solvents and ionic liquids are known for their physicochemical properties, such as high ionic conductivity, tunability, and environmental friendliness.^20,21^ These features make them ideal for enhancing the electrochemical stability and selectivity of biosensors. Hydrogels, on the other hand, offer excellent flexibility, high water retention, and biocompatibility, making them suitable for creating interfaces between biological tissues and electronic components.^22^

**Figure 1:**
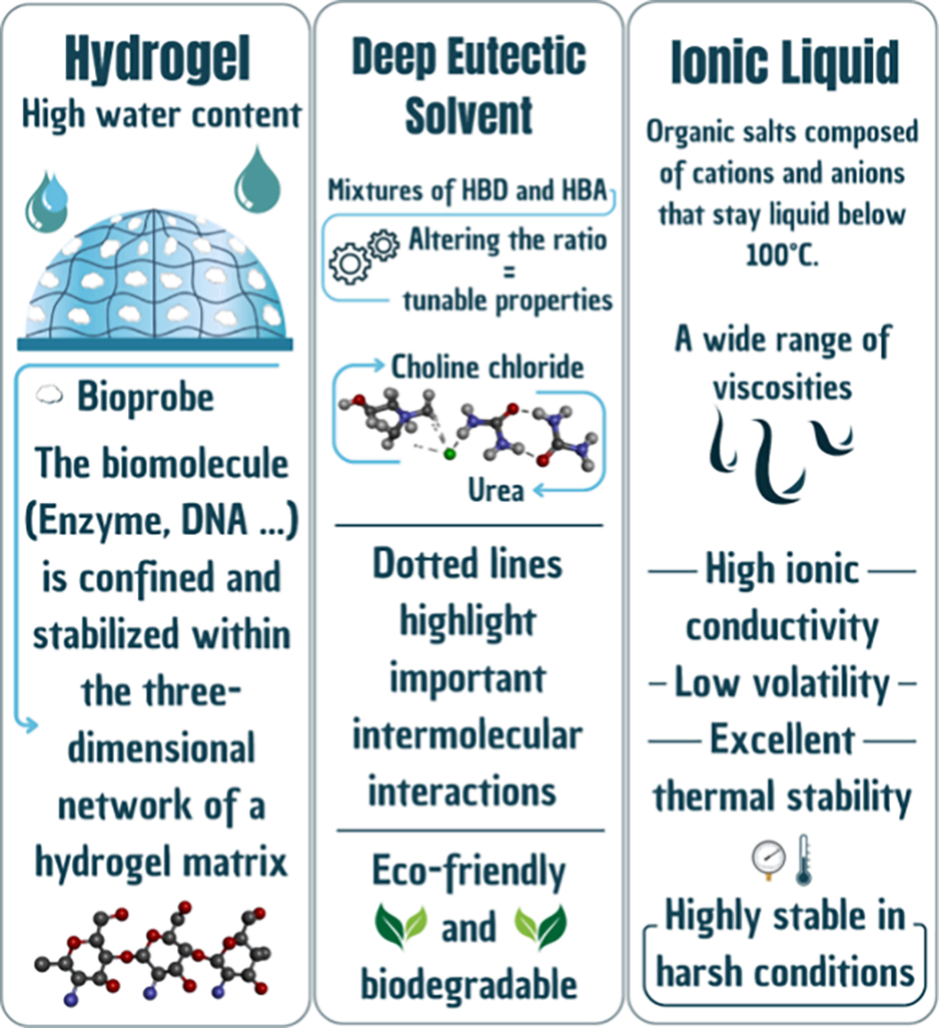
Comparison of the main properties of hydrogels, deep eutectic solvents (DES), and ionic liquids (ILs) used in enzymatic biodispositives.

Hydrogels are defined by IUPAC as networks of hydrophilic polymer chains that can absorb and retain significant amounts of water while maintaining their structural integrity.^23^ This characteristic is highly relevant to biosensor applications because hydrogels create an aqueous environment that is crucial for maintaining the bioactivity of enzymes.^24^ In enzyme-based biosensors, hydrogels serve as excellent platforms for enzyme immobilization, preserving their functionality by offering a moist, biologically compatible environment.^25,26^ Their ability to swell without dissolving ensures that enzymes can continue catalyzing reactions while analytes diffuse through the hydrogel matrix to reach the active enzyme sites.^27^ This makes hydrogels especially valuable in medical and biological applications, where they interface directly with tissues or bodily fluids. Furthermore, the cross-linked polymer structure contributes to the durability and stability of the biosensor, ensuring it remains functional over long periods, even in dynamic conditions such as inside the human body^28^

Ionic liquids (ILs) are defined as salts that remain in a liquid state at temperatures below 100 °C. As a result, the ions are more disordered in these cases compared to those in inorganic salts, for instance. This definition emphasizes their ionic nature, which gives ILs interesting properties such as high ionic conductivity, low volatility, and excellent thermal stability.^29–31^ These characteristics make ILs particularly useful in electrochemical bioelectronics, where efficient electron transfer is essential for generating accurate signals. The ionic structure of ILs allows for the movement of charged species, facilitating interactions between enzymes and electrodes. This enhances the sensitivity and responsiveness of the biosensor, enabling it to detect even small concentrations of analytes. Additionally, ILs are known for their ability to stabilize enzymes in harsh environments, such as high temperatures or extreme pH conditions, which extends the operational lifespan of a biosensor.^32^ Alternative approaches to enhance enzyme immobilization and electron transfer include the use of nanostructured materials, such as mesoporous frameworks formed by magnetically induced assembly of nanoparticles. These structures have demonstrated significant improvements in electrochemical response, particularly for small redox proteins.^33^ The tunability of ILs, through the selection of different cations and anions, allows for the customization of their physical and chemical properties, making them adaptable for various applications.^34,35^ However, this versatility also presents challenges, as certain IL formulations can be highly viscous or toxic, limiting their use in some biological or medical applications. Despite these challenges, ILs offer significant advantages in biosensors that require long-term stability and high sensitivity, particularly in industrial and environmental contexts.

This review provides a comparative analysis of the roles of these materials in enzyme-based biosensors and bioelectronics, including microbatteries and bioelectrosynthesis, highlighting their respective strengths, limitations, and future opportunities. The integration of these materials holds great potential for advancing bioelectronics technologies, with applications spanning medical diagnostics, environmental monitoring, and industrial processes. By addressing current challenges and optimizing these materials for large-scale use, the future of enzyme-based devices could see significant improvements in efficiency, sensitivity, and sustainability.

## 1 Why Hydrogels, DES, and ILs

Table 1 presents a comparative overview of the key characteristics of hydrogels, DES, and ILs, highlighting their chemical composition, structural properties, and applicability in enzyme-based bioelectronics devices. Hydrogels are composed of polymer networks with a high-water content, which makes them highly biocompatible and suitable for mimicking biological environments.^36–38^ These networks can be made from natural polymers such as alginate, chitosan, or collagen, or from synthetic polymers like polyethylene glycol (PEG) and polyacrylamide.^39–43^ For instance, a recent study demonstrated that carboxymethyl hemicellulose hydrogels integrated with nitrogen-doped carbon dots (CM-Hemi@Ca–N–CDs) exhibited significant antibacterial and antifungal activity.^44^ These hydrogels effectively inhibited the growth of *Escherichia coli*, *Staphylococcus aureus*, and *Candida albicans*, with enhanced antimicrobial performance attributed to strong binding interactions and improved structural rigidity facilitated by amide bond formation. In contrast, DES are mixtures of hydrogen bond donors (HBDs) and hydrogen bond acceptors (HBAs), typically formulated from simple and nontoxic components like choline chloride and urea.^17^ DES are characterized by their ability to create a liquid phase with melting points lower than those of the individual components.^45^ On the other hand, ILs are composed of organic salts formed from cations and anions, such as imidazolium, pyridinium, or phosphonium-based molecules.^46–48^ This structure gives ILs their excellent ionic conductivity and thermal stability, making them highly suitable for electrochemical applications.

**Table 1:** Comparative Table Focusing on the Chemical Aspects and Types of Hydrogels, Deep Eutectic Solvents (DES), and Ionic Liquids (ILs), Used in Enzyme-Based Biosensors

	hydrogels	DES	ILs
chemical composition	polymer networks with high water content	mixtures of hydrogen bond donors (HBDs) and hydrogen bond acceptors (HBAs)	organic salts composed of cations and anions
common types	natural (e.g., alginate, chitosan, collagen)	type I (choline chloride + + zinc chloride)	imidazolium-based (e.g., 1-butyl-3-methylimidazolium)
	synthetic (e.g., polyacrylamide, polyethylene glycol)	type II (choline chloride + chromium(iii) chloride hexa-hydrate)	pyridinium-based (e.g., N-butylpyridinium bromide)
	hybrid hydrogels (natural + synthetic)	type III (choline chloride + ethylene glycol)	phosphonium-based (e.g., trihexyl(tetradecyl)phosphonium)
		type IV (zinc chloride + urea)	
		type V (thymol + menthol)	
cross-linking methods	physical (ionic or hydrogen bonds, entanglements)	covalent or noncovalent interactions between HBDs and HBAs	van der Waals forces, hydrogen bonding, electrostatic interactions
	chemical (covalent bonds via initiators)		
functional groups	carboxyl (−COOH) and hydroxyl (−OH)	carboxyl (−COOH), hydroxyl (−OH) and carbonyls (-C = O)	amine (−NH_2_), thioether (-S-), fluoroalkyl (−CF_3_), and silane (-SiH_3_)
water affinity	high water absorption and retention	low to moderate water affinity, depending on composition	Hydrophobic or hydrophilic, depending on cation/anion pair
types of polymerization	radical polymerization	self-assembled mixtures (no polymerization)	no polymerization, based on ionic interactions
viscosity	low to moderate (depending on cross-linking density)	tunable from low to high viscosity	varies widely (low to high, depending on composition)
chemical stability	stable under mild conditions	chemically stable, tunable by modifying HBDs and HBAs	highly stable, resistant to high temperatures and harsh conditions
applicability	bioelectronics, biosensing, and tissue engineering; ideal for interfacing with biological systems	extraction, enzyme stabilization, and soft electrolyte matrices in biosensors	electrochemical (bio)sensors and extraction; improve stability, selectivity, and device performance

In terms of common types, hydrogels are classified into natural, synthetic, and hybrid forms. Natural hydrogels, such as alginate or collagen-based gels, are often preferred for biocompatibility, while synthetic versions like PEG offer greater control over mechanical properties. DES are typically classified into five types: Type I involves a quaternary ammonium salt combined with a metal chloride (e.g., choline chloride + zinc chloride),^49^ Type II composed of a quaternary ammonium salt and a metal chloride hydrate (e.g., choline chloride + chromium­(iii) chloride hexa-hydrate),^50^ Type III formed by a quaternary ammonium salt and a HBD (e.g., choline chloride + ethylene glycol),^51^ type IV that involves the interaction of a metal chloride and a HBD (e.g., zinc chloride + urea),^52^ and type V composed only of molecular substances (e.g, thymol + menthol).^53^ ILs are categorized based on their cation type, including imidazolium-based ILs (e.g., 1-butyl-3-methyl-imidazolium), pyridinium-based ILs, and phosphonium-based ILs, each providing different electrochemical characteristics suited for specific bioelectronics applications.^54^

The cross-linking methods used in hydrogels include both (such as ionic or hydrogen bonds and entanglements)^55^ and chemical cross-linking through covalent bonds initiated by external agents.^56^ In contrast, DES rely on covalent or noncovalent interactions between HBDs and HBAs to form stable mixtures, without requiring polymerization;^57^ However, it is worth noting that DES are polymerizable and, in such cases, useful for the production of, for example, eutectogels and hydrogels can be used for drug delivery, as deformation sensors, and in various components of flexible electronics.^58–60^ ILs, on the other hand, maintain their structure through van der Waals forces, hydrogen bonding, and electrostatic interactions, providing excellent chemical stability even in harsh conditions.^61^

Discussing the functionalization of these materials is important because functional groups control how enzymes are immobilized in a bioelectronics, directly impacting its effectiveness and performance. Hydrogels contain functional groups like carboxyl (−COOH) and hydroxyl (−OH), forming three-dimensional networks that allow for enzyme retention and stability, which are essential for sensor efficiency, for example.^62^ On the other hand, the functionalization of DES is based on interactions between HBD and HBA. This type of material is less dependent on covalent or ionic bonds, as in ILs, and its properties are strongly influenced by the proportion of components and water content. Common functional groups in DES include carboxylic acids, hydroxyl groups, and carbonyls (-C = O).^63^ ILs, in turn, can be modified with functional groups such as amine (−NH_2_), thioether (-S-), fluoroalkyl (−CF_3_), and silane (-SiH_3_) groups to tailor their physicochemical properties for specific applications, such as CO_2_ capture or catalysis; this functionalization can alter surface tension and molecular orientation at the interface, crucial aspects for enzymatic activity in the device.^64,65^

Regarding viscosity, hydrogels generally exhibit low to moderate viscosity, which can be adjusted by altering the cross-linking density.^66^ DES offer tunable viscosity, ranging from low to high, depending on the chosen HBD-HBA pair.^67^ ILs show a wide range of viscosities, from low to high, which can be customized by modifying the composition of the ionic pairs, with a range of viscosity between 10 and 10,000 mPa s.^68^ Chemical stability is another key factor in the suitability of these materials for bioelectronic applications. Hydrogels are typically stable under mild conditions, which makes them ideal for biological applications.^26,69^ DES are chemically stable and can be tuned by modifying the components involved in their formation.^70^ ILs, however, are highly stable and resistant to extreme temperatures and harsh conditions, making them particularly advantageous in industrial and high-performance biosensor environments.^71,72^

## 2 Hydrogels

Hydrogels have garnered significant attention in the field of enzyme-based biosensors, bioelectronics and biomimetic devices due to their unique properties and the ability to serve as effective platforms for enzyme immobilization and as interfaces for electrochemical process in bioelectronics systems. These materials are defined by their high-water content, biocompatibility, and mechanical flexibility, making them ideal for interfacing with biological systems in various biomedical and biosensing applications.^72–75^ Hydrogels are composed of three-dimensional polymer networks that can retain large amounts of water, which contributes to their biocompatibility and mimics the aqueous environment found in biological tissues.^76^ This feature, coupled with their capacity to encapsulate enzymes while preserving their bioactivity, makes hydrogels an ideal medium for biosensors.

One of the most important properties of hydrogels is their high-water content. This characteristic allows them to provide a moist environment like that of biological tissues, which is essential for maintaining the activity and stability of enzymes.^77^ Enzymes are biological catalysts that typically require aqueous conditions to perform optimally, and hydrogels offer the necessary hydration and mobility for substrates and enzymes to interact effectively.^78^ Moreover, the high-water content contributes to the hydrogel’s porosity, which facilitates the diffusion of analytes and enhances reaction kinetics.

Hydrogels also exhibit biocompatibility, a crucial factor for any material used in biosensors intended for biological applications. Biocompatibility ensures that the material does not induce adverse reactions when in contact with living tissues or enzymes, thus preserving the functionality of the biosensor over time.^79,80^ Hydrogels are often made from naturally derived polymers such as alginate, chitosan, or collagen, or from synthetic polymers like polyethylene glycol (PEG), which further contributes to their compatibility with biological systems.^81^ This makes them suitable for long-term use in biosensors, particularly in in vivo applications where interaction with tissues or bodily fluids is required.^82^ Another key property of hydrogels is their mechanical flexibility. Hydrogels are soft and deformable, which allows them to adapt to different shapes and surfaces, making them useful in applications requiring flexibility, such as wearable or implantable biosensors. This mechanical adaptability also enables hydrogels to maintain structural integrity under stress, which is vital for ensuring the stability and durability of the biosensor in dynamic environments, such as inside the human body.^83–85^

Hydrogels play a pivotal role in enzyme encapsulation, which is one of the primary reasons for their use in enzyme-based biosensors. This encapsulation involves embedding enzymes within the hydrogel matrix, forming a protective microenvironment that shields them from environmental stressors such as temperature fluctuations, pH changes, or exposure to organic solvents, all of which could lead to denaturation or loss of activity. Moreover, the highly hydrophilic nature of hydrogels not only preserves enzyme bioactivity but also enhances stability by maintaining an aqueous environment conducive to enzymatic reactions.^25,86,87^ For instance, Figure 2a outlines the formation of dZIF-8 BH biohybrid hydrogel in its forms of beads, sheets and fibers. These hydrogels combine the structural support of defective ZIF-8 frameworks with the stretchability and water retention properties of alginate-based hydrogels. This synergy not only extends the stability of encapsulated glucose oxidase (Figure 2b), but also enhances catalytic performance by facilitating substrate accessibility and efficient product accumulation.^86^

**Figure 2:**
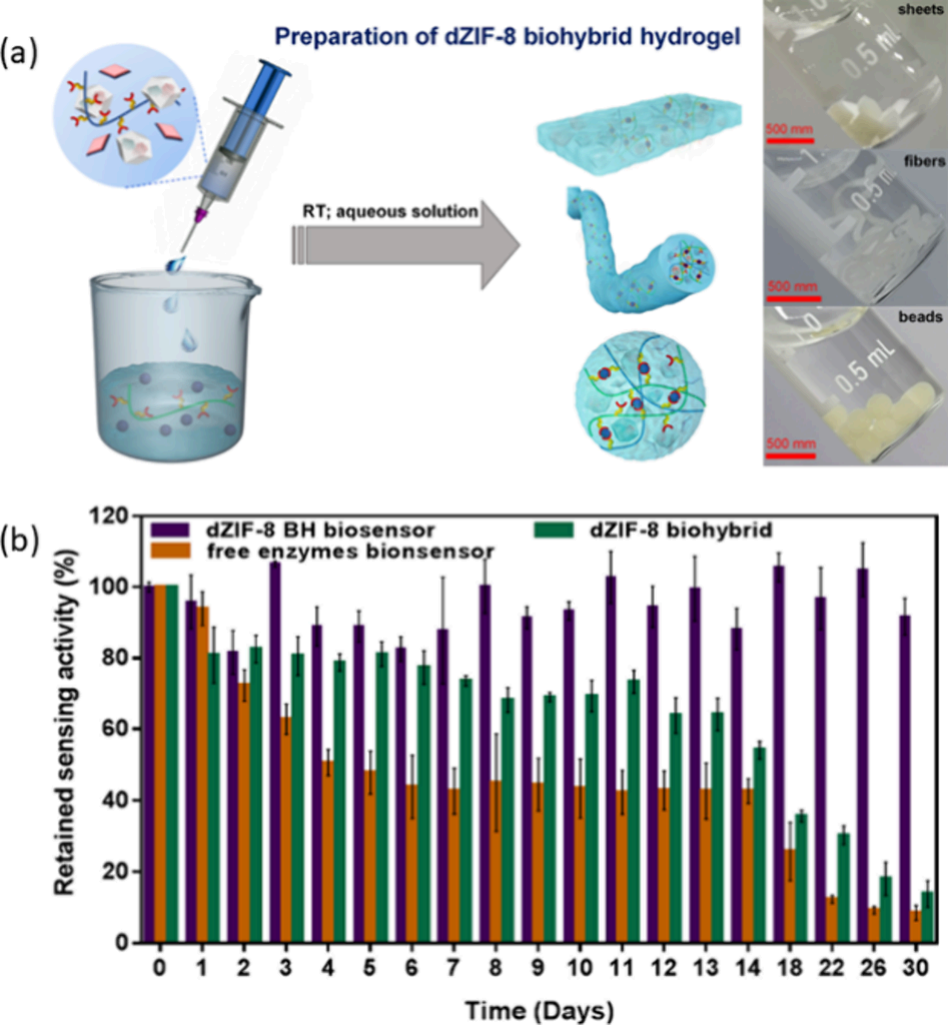
(a) Illustration of the preparation process of dZIF-8 BH through double-cross-linked alginate gelatinization and its application in colorimetric sensing based on a biocatalytic cascade mechanism; (b) retained sensing activity of biosensors stored at room temperature for 30 days highlights the stability of the system under testing conditions (1 mM glucose, 15 min incubation). Reprinted from ref 86 with permission from the American Chemical Society, copyright 2022.

In addition to enzyme encapsulation, hydrogels facilitate the transport of analytes, which is critical for the operation of biosensors. Due to their porous structure, hydrogels allow the diffusion of analytes into the matrix, where they can interact with the immobilized enzymes.^88,89^ This diffusion is essential for biosensors to detect specific molecules, such as glucose or lactate, in real time. The transport of analytes through the hydrogel matrix enhances reaction kinetics by enabling rapid interaction between the analyte and the enzyme, which is vital for generating a detectable signal in a timely manner.^90^ This property makes hydrogels particularly suitable for biosensors that require fast response times, such as glucose monitoring in diabetic patients or lactate sensing during exercise.

The incorporation of enzymes into gels provide a highly structured and stable microenvironment for bioelectrocatalysis. Figure 3 illustrates the one-pot synthesis of the BOD-based biogel, where Nafion and glutaraldehyde facilitate enzyme entrapment while preserving its catalytic function (Figure 3a). The resulting biogel is then integrated into a gas diffusion electrode (GDE), forming a composite structure that enhances enzyme retention and electron transfer (Figure 3b). To assess the structural properties of the biogel, optical microscopy images of the enzyme film reveal a homogeneous yet porous morphol ogy, ensuring efficient substrate diffusion (Figure 3c). Additionally, FTIR analysis confirms the successful incorporation of BOD and Nafion through characteristics vibrational bands (Figure 3d), further supported by chemical imaging that maps the enzyme and polymer distribution across the electrode surface (Figure 3e). Electrochemical characterization by cyclic voltammetry (CV) highlights the catalytic performance of the BOD-based biogel. In the absence of the O_2_, the biogel electrode exhibits minimal faradaic response, similar to the enzyme-free control (Figure 3f). However, under oxygen-saturated conditions, a significant increase in catalytic current is observed (Figure 3g), confirming the efficient bioelectrocatalytic activity of the immobilized enzyme. These results underscore he biogel’s effectiveness in stablilizing the enzyme while enabling direct electron transfer, a key feature for next-generation biosensors and bioelectrodes.^91^

**Figure 3:**
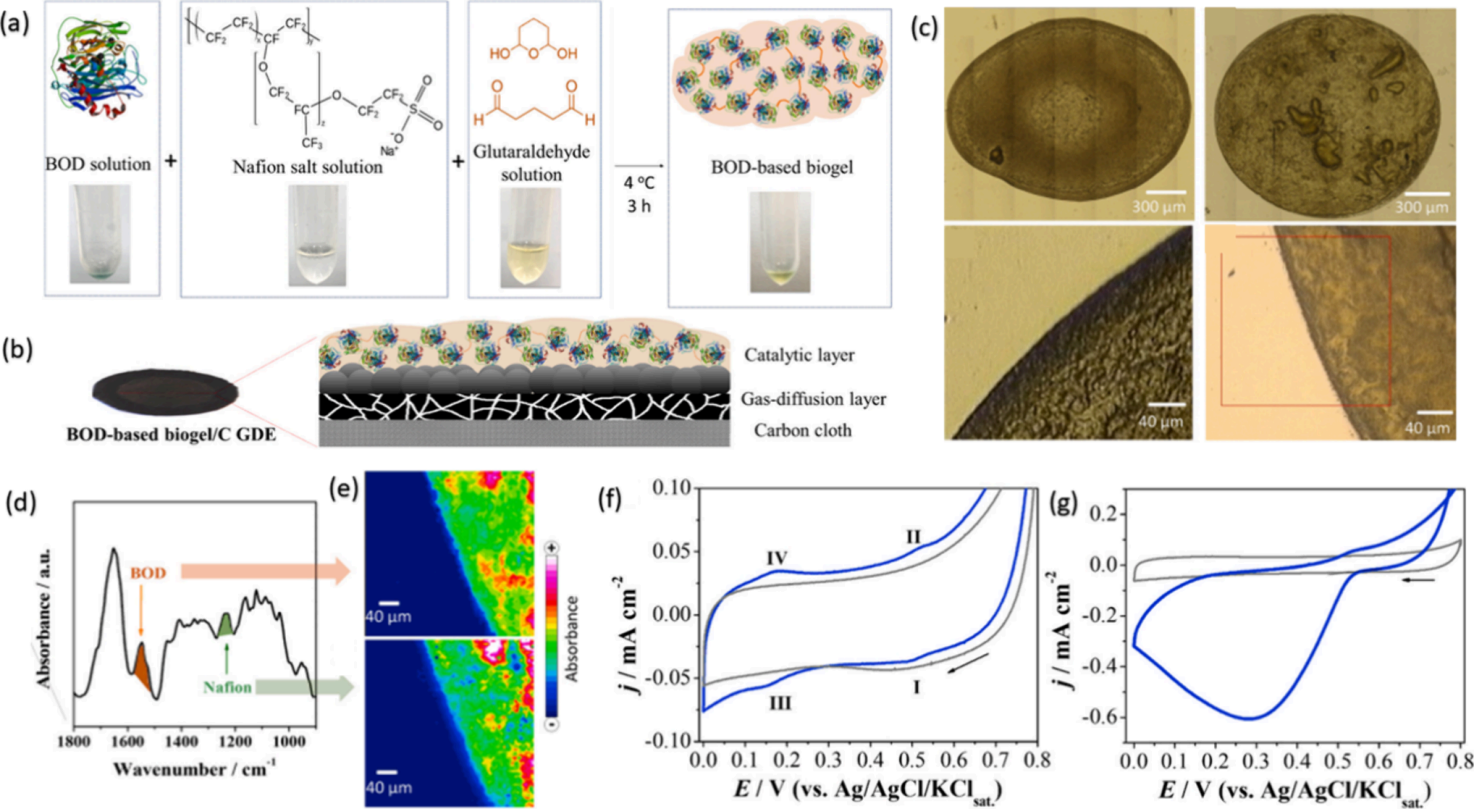
(a) One-pot synthesis of the BOD-based biogel; (b) schematic representation of the BOD-based biogel integrated into a gas diffusion electrode (GDE); (c) Optical images of the enzyme film; (d) FTIR spectrum confirming the incorporation of BOD and Nafion; (e) chemical mapping of characteristic vibrational bands at 1543 cm^–1^ (amide-II, BOD) and 1233 cm^–1^ (CF_2_ asymmetric stretching, Nafion); (f) cyclic voltammograms (CVs) in phosphate buffer (pH 7.2, 25 °C) comparing BOD-based biogel (blue) and enzyme-free electrode (gray) under anaerobic conditions; (g) CVs under oxygen-saturated conditions highlighting the bioelectrocatalytic activity of the immobilized enzyme. Reprinted from ref 91 with permission from the Elsevier, copyright 2021.

Hydrogels have found widespread use in various biosensing applications, particularly in glucose and lactate biosensors. Glucose biosensors, for example, are commonly used in the management of diabetes, where accurate and continuous monitoring of glucose levels is critical for patient care. In these biosensors, glucose oxidase, the enzyme responsible for catalyzing the oxidation of glucose, is encapsulated in a hydrogel matrix.^26,92–97^ Similarly, lactate biosensors, which are used in sports and medical diagnostics to monitor lactate levels in the body, utilize hydrogels to encapsulate lactate oxidase, facilitating the detection of lactate in real time.^97–99^ In both cases, hydrogels enhance the stability and performance of the enzyme-based biosensor, making them essential components of these devices. The use of hydrogels in enzyme-based biosensors offers several advantages, starting with their high biocompatibility. Since biosensors often come into direct contact with biological tissues or fluids, it is essential that the materials used do not induce harmful reactions or degrade in such environments. Hydrogels, particularly those made from natural polymers, are inherently biocompatible, allowing for safe and prolonged use in biosensing applications without eliciting immune responses or toxicity.^85,100,101^

Despite their many advantages, hydrogels have certain limitations when used in enzyme-based biosensors. One of the primary drawbacks is their limited ionic conductivity. Since hydrogels are composed mainly of water and polymer, their ability to conduct ions is relatively low compared to other materials, such as ionic liquids or deep eutectic solvents. This can limit the efficiency of electron transfer processes in certain electrochemical biosensors, reducing the overall sensitivity and signal output of the device. These issues have been previously discussed in the literature, highlighting that enzymatic biosensors often suffer from rapid activity loss and nonspecific adsorption, limiting their commercial viability.^102^ To address this issue, researchers often modify hydrogels by incorporating conductive materials, such as inorganic salts^103,104^ to enhance their ionic conductivity.

Building on these efforts to overcome the inherent limitations of hydrogels, recent studies have explored innovative systems with enhanced electrochemical properties and mechanical stability. A notable example is the BEAQ-gel.^105^ Figure 4a shows that BEAQ-gel exhibit quasi-reversible behavior, highlighting its potential as a redox-active material for bioelectronics applications. Additionally, its mechanical stability (Figure 4b) at elevated temperatures ensures it suitability for wearable devices, maintaining structural integrity even under physiological conditions. Furthermore, the small-angle X-ray scattering (SAXS) analysis (Figure 4c) reveals a hierarchical internal structure that promotes ion diffusion and could enhance enzyme encapsulation. Complementary to this, Figure 4d presents the electron density profile obtained via SAXS, providing further insight into the organized supramolecular nature of BEAQ-gel. This well-structured network facilitates ion transport, which is crucial for maintaining electrochemical performance in bioelectronics applications. Beyond its electrochemical properties, the versatility of BEAQ-gel extends to its integration into functional devices. Figure 4e illustrates the structural design of a wearable microbattery using BEAQ-gel, demonstrating its adaptability for flexible and stretchable applications. Moreover, Figure 4f compares the battery with a commercial CR2032 cell, highlighting its compact size and flexibility. These features make BEAQ-gel a promising material for energy storage applications in wearable technologies.

**Figure 4:**
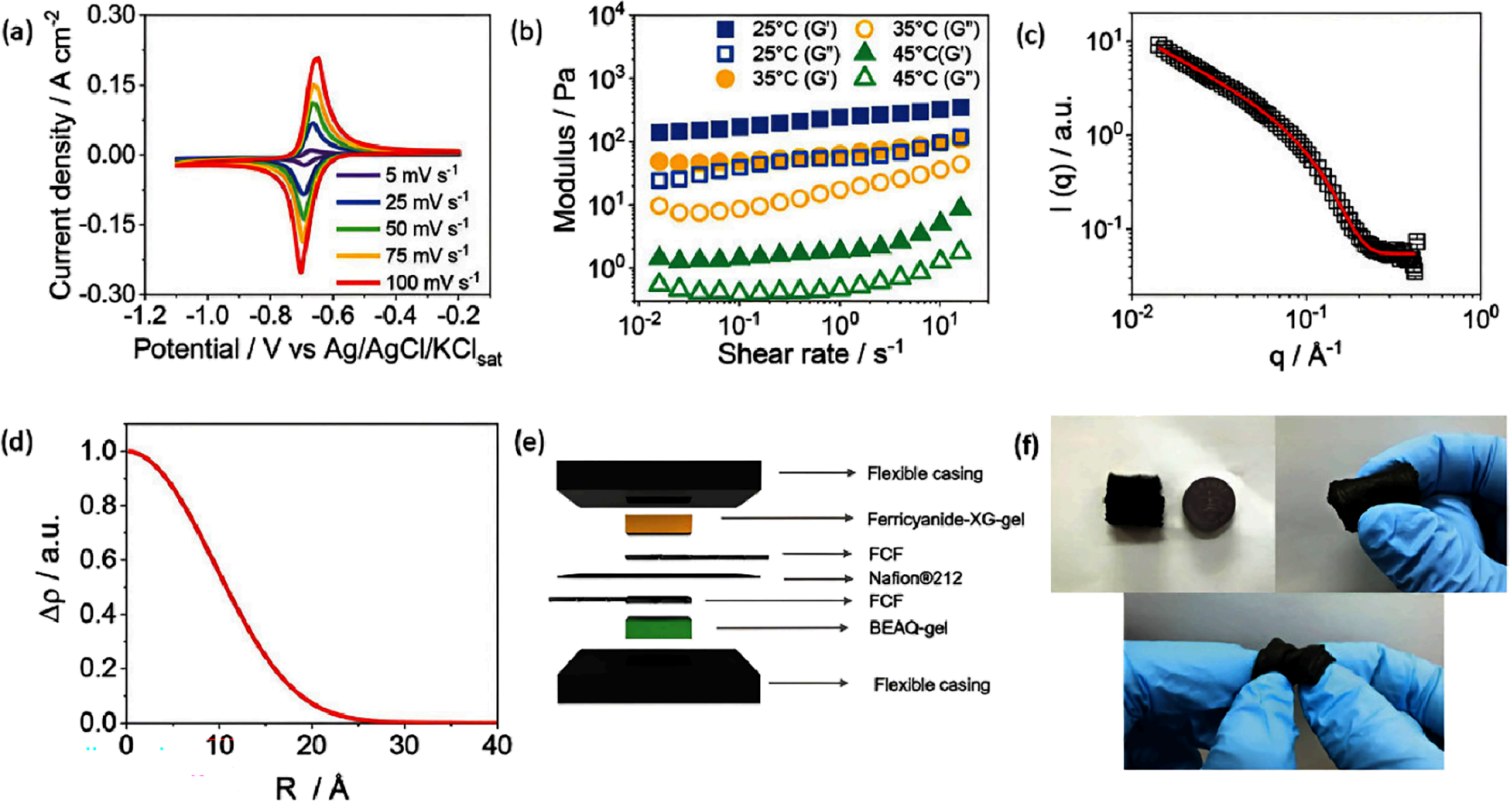
(a) CVs of the BEAQ-gel at different scan rates using flexible carbon fiber as working electrode. (b) Mechanical spectra of BEAQ-gel (3% w/v) at various temperatures; (c) small-angle X-ray scattering (SAXS) profile of the BEAQ-gel; (d) electron density profile derived from SAXS data; (e) schematic representation of the wearable microbattery integrating the BEAQ-gel; (f) optical images of the BEAQ-gel. Reprinted from ref 105 with permission from the John Wiley and Sons, copyright 2024.

Hydrogels are also sensitive to environmental changes, such as variations in pH and temperature.^106^ Since the structure and function of enzymes are highly dependent on environmental conditions, changes in the surrounding environment can lead to alterations in the hydrogel’s structure or enzyme activity. For instance, extreme pH shifts may cause the hydrogel to swell^107^ or shrink,^108^ which could disrupt the encapsulated enzyme’s activity or even lead to leaching of the enzyme from the matrix. Similarly, temperature fluctuations could affect both the hydrogel’s mechanical properties and the enzyme’s catalytic efficiency, leading to decreased performance in biosensing applications.

## 3 Deep Eutectic Solvents (DES)

DES are formed by mixing two or more components, typically a hydrogen bond donor (HBD) and a hydrogen bond acceptor (HBA), that interact through hydrogen bonding, resulting in a liquid phase with melting points lower than those of the individual components.^45^ DES have emerged as a promising class of materials for enzyme-based bioelectronics due to, for example, their ability to solubilize a wide range of molecules thanks to a network of hydrogen bonds favored by the presence of HBDs and HBAs.^109^ Furthermore, DES are a promising low cost class of materials with simple synthesis and unique properties, which include low toxicity and biodegradability.^110^ These characteristics, added to their ability to enhance the stability and activity of enzymes while improving the electrochemical properties,^17,111^ make DES an attractive alternative to conventional solvents and ionic liquids, especially in the context of green chemistry and sustainability, garnering significant attention to DES in recent years.^112–115^ For example, the development of a catalase-based biosensor for hydrogen peroxide detection, employing a poly­(safranine T) film electropolymerized in a ternary DES system, resulted in a remarkably low detection limit of 34 nM, outperforming many previously reported enzyme-based biosensors.^111^ This biosensor also exhibited high selectivity against common interferents such as glucose, uric acid, ascorbic acid, and dopamine, as illustrated in Figure 5a. Moreover, long-term stability tests showed that after one month of storage at 4 °C, the sensor retained 60% of its initial response (Figure 5b), indicating a promising durability profile

**Figure 5:**
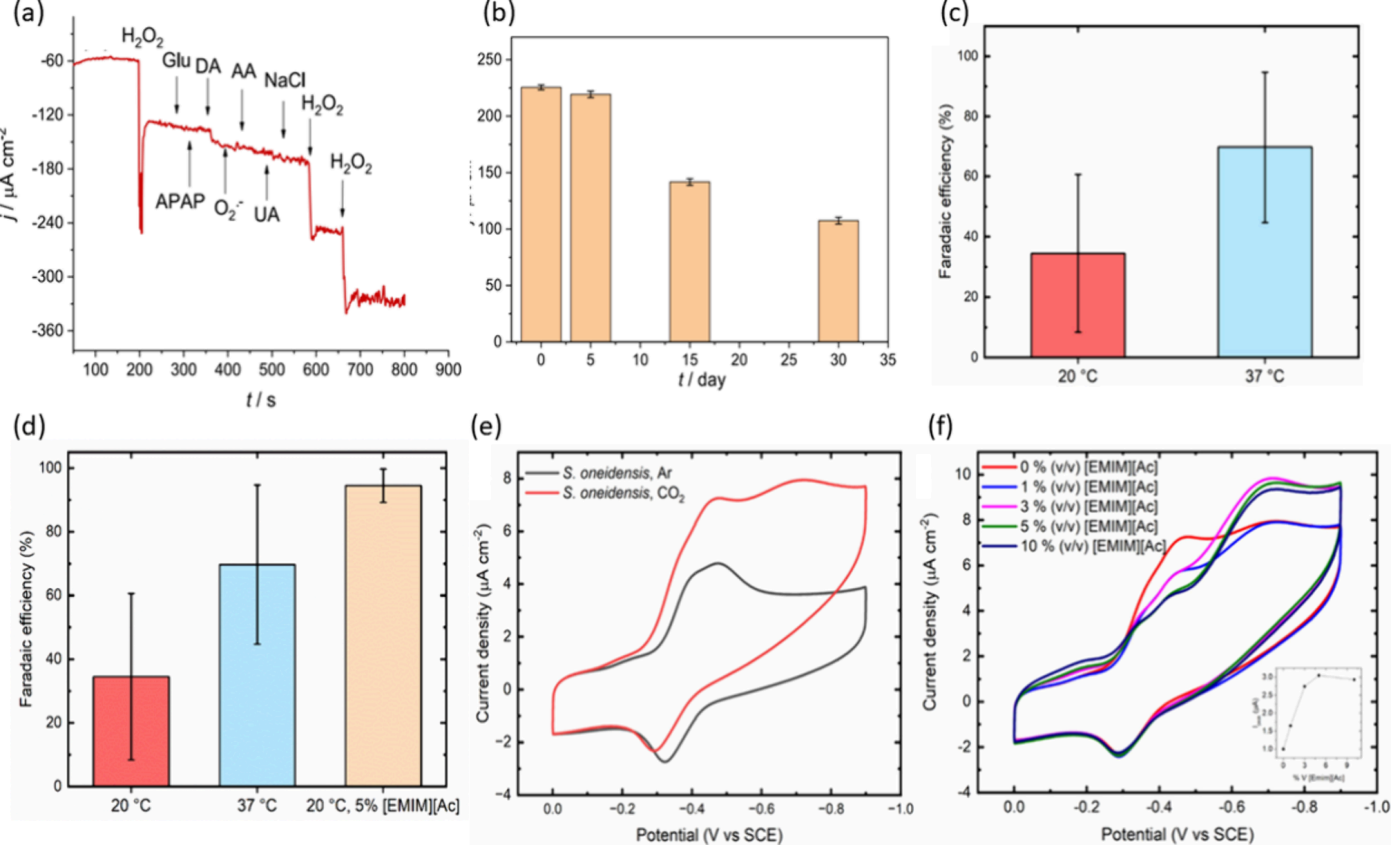
(a) Current response at −0.2 V vs Ag/AgCl in 0.1 M NaPB (pH 7.0) at CAT/PSF/MWCNT/GCE, recorded after successive additions of H_2_O_2_ in the presence of various interferents; (b) stability of the biosensor over time, retaining 95% of its initial response after 5 days, 78% after 15 days, and 60% after 30 days. Reprinted with permission under a Creative Commons CC BY License from Razieh Seyfi Zouleh et al., *Microchemical Journal*, 190, 108155 (2023). Copyright 2023 Elsevier; (c) Faradaic efficiency of CO_2_ reduction at 20 and 37 °C after 16 h of electrolysis; (d) comparison of formate production efficiency under different conditions; (e) cyclic voltammetry illustrating bioelectrocatalytic activity of MR-1 toward CO2; (f) Effect of [EMIM]­[Ac] concentration on MR-1 electrocatalytic performance toward CO_2_. Reprinted from ref 145 with permission from the American Chemical Society, copyright 2024.

One of the key properties that make DES suitable for enzyme-based bioelectronics systems is their low toxic and biodegradable nature. Unlike some traditional solvents and ionic liquids, which may pose environmental or health risks, DES are often composed of naturally occurring or easily accessible components, such as choline chloride and urea.^116^ This composition ensures that DES are safe for biological applications, making them ideal for biosensors used in medical diagnostics, environmental monitoring, and food safety.^117^ Overall, although DES offer several advantages over conventional solvents, such as reduced toxicity, it is essential to exercise caution in their use and to pursue ongoing research into any potential risks.^118^

Although some DES exhibit adequate ionic conductivity for electrochemical applications,^119^ this conductivity strongly depends on molecular reorientation dynamics and glassy behavior near the glass transition temperature (Tg).^120^ Table 2 provides the ionic conductivity values of several DESs across various temperatures. As the temperature increases, the viscosity decreases, allowing for greater ionic mobility and consequently raising the conductivity.^121^

**Table 2:** Ionic Conductivity Values of Several DESs across Various Temperatures

salts	HBD	ratio (mol:mol)	*T* (°C)	conductivity(mS cm^–1^)	ref
EtNH_3_Cl	acetamide	1:1.5	40	0.688	^179^
EtNH_3_Cl	urea	1:1.5	40	0.348	^179^
ChCl	CF_3_CONH	1:2	40	0.286	^179^
ChCl	urea	1:2	40	0.199	^179^
ChCl	urea	1:2	45	0.205	^180^
ChCl	urea	1:2	50	0.283	^180^
ChCl	urea	1:2	55	0.379	^180^

The ionic conductivity of DES is generally low (less than 2 mS cm^–1^ at room temperature) due to the high viscosity of these solvents. The viscosity, which hinders the free movement of ions, results from the extensive hydrogen bonding network and with other interactions like van der Waals and electrostatic forces among the components of the DES. Table 3 organizes the viscosity of a series of DES. As observed, increasing the temperature decreases the viscosity, and increases the ionic conductivity of DESs. This can be explained by the hole theory, which states that an ion can move in an ionic liquid only when it is near a hole of equal or larger size.^122^ DES have high viscosity and low conductivity because the average size of an ion is around 0.4 nm, which is twice the average radius of a hole.^52^ As a result, only a small fraction of ions is in motion at any given time due to the lack of appropriately sized gaps. On the other hand, both viscosity and ionic conductivity of DES are tunable, meaning that their properties can be adjusted by modifying the ratio of HBD to HBA or by adding other components, like acids, salts, and amine derivatives.^123^ This tunability allows for the customization of DES to suit specific applications, where viscosity can impact biomolecule mobility, reaction rates, and overall dispositive performance.

**Table 3:** – Viscosity Values of Several DESs across Various Temperatures

			viscosity (*cP*)				
			*T* (°C)				
salts	HBD	ratio (mol:mol)	40	45	50	55	ref
ChCl	ethylene glycol	1:2	31	29	27	24	^181^
ChCl	glycerol	1:2	104	82	64	52	^181^
ChCl	urea	1:2	231	161	119	95	^181^
ChCl	urea	1:1.5	365	255	181		^182^
ChCl	urea	1:2	443^a^			149.80^a^	^183^

a Viscosity measured for a system containing ChCl:Urea (1:2) + LiCl (0.419 mol/kg).

DES have demonstrated the capacity to enhance enzyme stability under a variety of challenging conditions. The hydrogen-bonding networks characteristic of DES reduce water activity and create a stabilizing microenvironment that mitigates enzyme denaturation and prolongs enzymatic activity.^124^ For instance, that natural deep eutectic solvents (NADES), such as betaine-sorbitol-water (1:1:3), significantly improve the thermal stability and catalytic activity of lipoxygenase (LOX) from Pleurotus sapidus, with activity increases of up to 43% compared to aqueous buffers.^125^ These features expand the usability of enzymes in industrial and biosensing applications under harsh conditions. Beyond their role in enzyme stabilization, DES also improve the performance and longevity of coenzymes such as NAD/NADH, which play pivotal roles in redox enzymatic reactions. Research has shown that choline chloride:urea DES significantly prolong the stability of these coenzymes, maintaining their functionality for up to 50 days.^112^ This effect is attributed to the structured hydrogen-bond network within DES, which reduces degradation pathways typically encountered in aqueous environments. Such stabilization is crucial for the accurate operation of biosensors in diagnostic and analytical applications,^126^ where the integrity of coenzymes directly impacts the precision of measurements.

Furthermore, DES are versatile solvents that can facilitate the synthesis of novel materials for biosensor construction. By leveraging their tunable properties, researchers can optimize their role in the formation of composites for electrode modification. For example, DES has been used to synthesize polyaniline nanocomposites,^127,128^ which have demonstrated enhanced electrochemical properties, making them ideal for biosensors,^129–134^ both for the immobilization of biomolecules and as electron mediators during the enzymatic reaction process.

This class of materials offer significant advantages for enzyme-based bioelectronics systems, including their ability to stabilize proteins and enzymes, enhancing their durability and catalytic efficiency, in addition to being a bet particularly in the context of green chemistry and sustainability. However, despite their benefits, DES face technical challenges, including high viscosity, which can hinder molecule diffusion and reduce mass transfer efficiency. The lack of comprehensive data on the long-term environmental impacts of large-scale DES disposal also remains a concern, emphasizing the need for further research on safety and sustainability^118^

## 4 Ionic Liquids (ILs)

The exploration of electron transport pathways in redox enzymes provides a deeper understanding of their integration into bioelectronics devices. Recent studies highlight the role of protein structures in facilitating electron transfer through localized conductance channels, as the conductance properties of bilirubin oxidase using advanced scanning tunneling microscopy techniques.^135^ Meanwhile, in situ and operando electrochemical techniques have enabled a deeper understanding of biomolecular electron transfer mechanisms, particularly in the context of redox proteins and bioelectrode development. Although these studies focus on fundamental aspects of charge transfer, their findings provide valuable insights that can be leveraged to enhance the design and performance of biosensors.^136^ In that regard, ILs have attracted significant interest in enzyme-based biosensors and bioelectronics thanks to its ability to modulate electron transfer reactions.^137^ Furthermore, this class of materials presents interesting properties for sensibility, such as low volatility, outstanding thermal resilience, and superior electrical conductivity, which allow them to function effectively as electrolytes or enzyme-stabilizing agents.^138^ Their key attributes—adjustable ionic conductivity, low volatility, and thermal stability—make them highly suitable for use in bioelectronics platforms that require reliable and long-term performance, especially in harsh environmental conditions. The tunability of ILs allows for specific tailoring to meet the needs of bioelectronics, whether for improving enzyme stability, enhancing electron transfer, or increasing sensor sensitivity.^139^

Electron transfer is a fundamental process underpinning various phenomena in physics, chemistry, and biology. Its role in sustainable energy solutions and the synthesis of value-added compounds aligns with the development of enzyme-based biosensors, where electron transfer mechanisms are critical for improving device performance and stability.^140^ That way, one of the most notable properties of ILs is their adjustable ionic conductivity, which is essential for facilitating efficient electron transfer.^141,142^ The ionic nature of these liquids allows for the conduction of electricity through the movement of ions, which is crucial in bioelectronics where the success of application relies also on electrochemical reactions. The conductivity of ILs can be fine-tuned by altering the cation–anion pair, enabling researchers to optimize their designs for specific applications.^143,144^ A recent study investigated the impact of 1-ethyl-3-methylimidazolium acetate ([EMIM]­[Ac]) as a cosolvent for the bioelectrochemical reduction of CO_2_ to formate by *Shewanella oneidensis* MR-1.^145^ In the absence of ILs, exhibited a faradaic efficiency of 34.5 ± 26.1% at room temperature, which increased to 69.7 ± 22.2% at 37 °C (Figure 5c). The addition of 5% (v/v) [EMIM]­[Ac] at room temperature further enhanced system performance, elevating the faradaic efficiency to 94.5 ± 4.3% (Figure 5d. Moreover, the cathodic current density surged from 1.57 ± 0.68 μA cm^–2^, in the absence of IL, to 10.50 ± 1.90 μA cm^–2^, (Figure 5 **e, f**), highlighting the role of [EMIM]­[Ac] in facilitating CO_2_ solubility and enhancing electron transfer within the microbial system.

ILs are also known for their low volatility,^146^ which makes them excellent for applications under high vacuum conditions because, unlike volatile substances (such as water or organic solvents), they do not evaporate. This makes them ideal for devices or sensors that need to operate stably at extremely low pressures, ensuring safety and efficiency.^147,148^ Unlike volatile organic solvents, ILs remain stable at elevated temperatures without significant loss of mass or degradation, which is particularly important in applications that require extended operational times or exposure to harsh environmental conditions.

The thermal stability of ILs has been highlighted as a critical factor in enhancing the performance of electrochemical biosensors. In a recent study, acetylcholinesterase (AChE) entrapped in in ILs retained 73% of its initial activity after thermal treatment at 55 °C for 20 min, whereas unprotected AChE lost 60% of its activity at temperatures between 42–48 °C.^149^ This thermal protection is attributed to the ability of ILs to create a biocompatible microenvironment, reducing the impact of thermal fluctuations on the enzyme’s active site. Such a property enables the biosensor to operate efficiently under elevated temperatures, facilitating the analysis of pesticides in real samples, such as fruits and vegetables, without the need for prior cooling, thereby improving process efficiency.

Additionally, in electrochemical biodispositives, ILs can act as the electrolyte due to their remarkable ionic conductivity, which typically ranges between 10^–3^ and 10^–2^ S cm^–1^ at room temperature, ensuring efficient ion transport and minimizing resistance.^150^ Their wide electrochemical stability window, often exceeding 4.0 V, supports a broad spectrum without degradation of the electrolyte.^151^ This makes ILs particularly advantageous for maintaining stability and performance during long-term operation, even in complex biological environments, where traditional electrolytes might degrade or lose efficacy. For example, imidazolium-based ILs have demonstrated biomimetic electron transfer capabilities by emulating the function of histidine residues in biological systems. This facilitates a concerted two-electron transfer, similar to natural enzymatic processes. Studies have shown that such ILs can enhance the electron transfer efficiency of flavin mononucleotide (FMN) when compared to conventional inorganic electrolytes. The increased peak currents observed in cyclic voltammetry reflect improved conductivity and redox behavior, attributed to the organized ionic structure of ILs.^152^ This organized ionic structure supports more efficient ion transport, crucial for enhancing the stability and performance of enzyme-based bioelectronics platforms.

Moreover, beyond their use as electrolytes, ILs have emerged as promising materials for enzyme immobilization and stabilization in biosensors due to their properties and versatility. The composition of ILs, based on different cations and anions, enables the modulation of interactions between enzymes and immobilization materials, enhancing catalytic efficiency, thermal stability, and operational resistance of immobilized enzymes.^153,154^ ILs have shown to significantly enhance enzyme stability by minimizing denaturation effects, particularly under nonconventional reaction conditions. This has been demonstrated in the development of a highly sensitive amperometric biosensor, where the immobilization of tyrosinase on a modified electrode with ILs contributed to improved enzyme performance and stability, allowing for accurate analysis of phenolic compounds such as catechol.^139^ These advances underscore the role of ILs as a strategic tool in the development of innovative and sustainable enzymatic technologies, including glucose,^155^ hydrogen peroxide,^156^ and alcohol^157^ detection devices.

Despite their many advantages, ILs also have certain limitations that must be considered when designing enzyme-based biosensors. One of the primary challenges is the high viscosity of some ILs, which can impede the diffusion of analytes and reduce the overall speed of the biosensor’s response. While the viscosity of ILs can be tuned to some extent, finding the right balance between viscosity and conductivity can be challenging, particularly for biosensors that require rapid analyte detection.

Another limitation is the potential toxicity of certain IL components. While many ILs are biocompatible, such as those synthesized from amino acids,^158^ some cation–anion combinations can be toxic to living organisms, which may limit their use in devices intended for medical or environmental applications. For example, ILs with imidazolium or pyridinium cations and highly fluorinated anions, such as [(CF_3_SO_2_)_2_N] and [PF_6_], exhibit increased toxicity.^159^ This is often attributed to their structural features that enhance hydrophobicity and membrane disruption potential. Researchers must carefully select ILs that are safe and nontoxic, particularly for biosensors designed for in vivo use or in applications where human exposure is a concern. Lastly, the cost of ILs can be relatively high compared to conventional solvents. The synthesis of ILs often requires specialized equipment and processes, which can increase the overall cost. While ILs offer significant performance benefits, the cost may be prohibitive for some applications, particularly in cases where the technology is intended for disposable or large-scale use.

## 5 Comparative Analysis of Hydrogels, DES, and ILs

A comparative analysis of these materials reveals distinct strengths and limitations in terms of how they stabilize enzymes, interact with biological systems, and perform in bioelectronics technologies. Table 4 summarize key properties, advantages, limitations, and applications of these class of materials for bioelectronics devices. Effective enzyme stabilization is fundamental to the success of enzyme-based biosensors, as it ensures that enzymes retain their catalytic activity over time, even under changing environmental conditions. Hydrogels excel in maintaining enzyme bioactivity in aqueous environments. The hydrated network within hydrogels mimics the natural biological setting where enzymes typically function, preserving their structure and catalytic capabilities for extended periods. However, hydrogels are sensitive to environmental factors, such as changes in temperature or pH, which can alter their structure, leading to potential swelling or shrinking. This sensitivity can affect the stability of the encapsulated enzymes, potentially reducing the biosensor’s performance in conditions that are less controlled or more dynamic.

**Table 4:** Hydrogels, Deep Eutectic Solvents (DES), and Ionic Liquids (ILs) in Enzyme-Based Biosensors, Summarizing Key Properties, Advantages, Limitations, and Applications

feature	hydrogels	deep eutectic solvents (DES)	ionic liquids (ILs)
key properties	high water content, biocompatibility, flexibility	biodegradable, tunable viscosity, and ionic conductivity	adjustable ionic conductivity, thermal stability, low volatility
enzyme stabilization	good in aqueous environments, sensitive to pH and temperature	enhances enzyme stability in harsh conditions	excellent for electron transfer, maintains enzyme structure in harsh conditions
biocompatibility	high biocompatibility, safe for in vivo use	biodegradable, generally biocompatible	variable depending on formulation, potential toxicity in some ILs
electrochemical properties	moderate ionic conductivity, limits performance	tunable ionic conductivity, improves sensor sensitivity	excellent electron transfer, enhances redox reactions
advantages	ease of enzyme immobilization, structural stability	green chemistry, improves enzyme stability and sensor sensitivity	enhances sensitivity and selectivity, excellent long-term stability
limitations	limited ionic conductivity, sensitive to environmental changes	scalability challenges, limited exploration in biological samples	high viscosity, potential toxicity, costly for large-scale use
applications in biosensors	glucose and lactate biosensors, implantable devices	cholesterol, uric acid, and other enzyme-based biosensors	glucose, alcohol, hydrogen peroxide sensors
cost	low to moderate	moderate, but scalability could affect cost	high (depends on type of IL)
scalability	high scalability for bioelectronics	moderate, still under development for large-scale use	limited by the high cost and complexity of synthesis
environmental impact	biodegradable, environmentally friendly	eco-friendly, biodegradable	some ILs may pose environmental risks, nonbiodegradable

DES offer a distinct advantage in enzyme stabilization, particularly under harsh conditions. Their chemical composition, often involving hydrogen bond donors and acceptors, creates an environment that can protect enzymes from or the denaturation caused by high temperatures or the presence of organic solvents. This makes DES ideal fo industrial applications where systems may be exposed to more extreme environments. Despite this advantage, the use of DES in complex biological samples remains less explored. Their interactions with biological matrices, such as blood or tissue fluids, need further investigation to ensure that they do not negatively impact enzyme activity or biosensor reliability.

ILs are particularly noted for their ability to enhance electron transfer, which is crucial for bioelectronics that rely on electrochemical signals. Their ionic nature supports efficient electron exchange, allowing for the rapid detection of analytes. However, ILs can present challenges in terms of enzyme stabilization due to their high viscosity in some formulations. This can hinder the diffusion of substrates and analytes to the enzyme, potentially slowing down the response time. Furthermore, certain ILs have raised concerns about toxicity, particularly when used in biological systems, limiting their application in some biosensor designs.

Biocompatibility is another critical factor for enzyme-based bioelectronics, especially those intended for in vivo applications or prolonged interaction with biological tissues and fluids. Hydrogels are highly biocompatible, making them suitable for implantable biosensors or sensors designed for direct contact with human tissue. Natural hydrogels, such as those derived from alginate or chitosan, are particularly valued for their minimal toxicity and ability to integrate seamlessly with biological systems. This allows for extended use in medical diagnostics or therapeutic monitoring without the risk of adverse reactions. However, synthetic hydrogels may require additional modifications to match the biocompatibility levels of their natural counterparts, particularly when used in sensitive biological environments.

DES are generally considered safe and biodegradable, making them appealing for applications that prioritize environmental sustainability. Their low toxicity and biodegradability, especially when derived from naturally occurring components like choline chloride, make DES ideal for applications in environmental monitoring or food safety. As interest in their use in biomedical applications grows, further research is needed to confirm their full biocompatibility across a wider range of biological systems. This includes understanding how DES interact with living cells and tissues over long periods, particularly for biosensors that may need to function in more complex or reactive environments.

ILs, while tunable in terms of their biocompatibility, present a more complex picture. The cation–anion combinations used in IL formulations can significantly influence their safety and toxicity profiles. Some ILs, particularly those based on imidazolium, have been found to exhibit cytotoxicity, which can restrict their use in medical or environmental biosensors. Moreover, ILs with long alkyl chains in the cation further amplify toxic effects by interacting strongly with biological membranes.^159,160^ However, other IL formulations, such as those based on phosphonium or pyridinium, have been developed with improved biocompatibility, allowing them to be used in biosensors designed for less invasive applications. Despite this, the variability in IL formulations necessitates careful selection to ensure that the material is safe for the intended use, especially in cases where human or environmental exposure is likely.

In terms of performance in bioelectronics, each of these materials offers unique advantages and limitations. Hydrogels are highly effective for enzyme immobilization, creating a stable environment that preserves enzyme activity while allowing analytes to diffuse through the matrix. Their porous structure supports efficient enzyme–substrate interactions, making hydrogels ideal for biosensors that operate in aqueous environments.^161^ However, their limited ionic conductivity poses a challenge in electrochemical biosensors, where efficient electron transfer is necessary for signal generation. This limitation can be mitigated by incorporating conductive materials, such as nanoparticles or carbon nanotubes, into the hydrogel matrix, though this increases complexity and cost.

DES are particularly valuable for improving enzyme stability and enhancing the overall. Their ionic nature contributes to improved electrochemical properties, facilitating electron transfer and boosting sensor performance. Additionally, DES offer significant eco-friendly benefits, being biodegradable and derived from relatively inexpensive and safe materials. These properties make DES a strong candidate for biosensors used in environmental monitoring, where sustainability and minimal environmental impact are key concerns. However, DES-based biosensors, for example, in complex biological environments still require further development to optimize their performance and stability when exposed to more unpredictable variables, such as bodily fluids or varying pH levels.

ILs excel in applications where enhanced electron transfer is critical for sensor performance. Their ionic nature allows for efficient movement of electrons, making them ideal for electrochemical systems that require high sensitivity and quick response times. ILs also offer long-term stability, ensuring that dispositive remain functional over extended periods without significant degradation of either the components or the enzymes involved.^138^ However, their high viscosity can impede the diffusion of analytes, limiting their effectiveness in sensors that require rapid detection or in applications where analytes are present at low concentrations. Additionally, concerns about the toxicity of certain IL formulations persist, particularly for biosensors designed for biomedical or environmental use, where leakage or degradation could pose risks.

In comparing these three materials, it becomes clear that hydrogels, DES, and ILs each offer distinct benefits depending on the application. Hydrogels are ideal for bioelectronics that require biocompatibility and enzyme immobilization in aqueous environments, though they face limitations in terms of ionic conductivity and sensitivity to environmental changes. DES provide excellent enzyme stabilization and enhanced electrochemical properties, making them a strong choice for eco-friendly dispositive, though their use in complex biological systems remains underexplored. ILs, with their outstanding electron transfer properties and a wide electrochemical stability window, are highly effective in electrochemical biosensors, though they are constrained by issues of viscosity and potential toxicity in certain formulations.

The future of enzyme-based bioelectronics may lie in the integration of these materials to capitalize on their respective strengths while mitigating their weaknesses.^162^ For instance, hybrid systems that combine the biocompatibility and stability of hydrogels with the enhanced electrochemical properties of DES or ILs could provide a balanced solution for a wider range of applications. Similarly, modifications to the chemical structure of DES or ILs could improve their performance in biological environments, expanding their utility in medical diagnostics, environmental monitoring, and industrial processes.

### 5.1 Where do Hydrogels, DES and ILs Stand in Biosensors, Bioelectronics, and Biomimetic Systems?

The overall performance of enzyme-based biosensors is not solely determinate by the intrinsic properties of hydrogel, DES, and ILs, but also by their spatial arrangement within the device. In this topic, we explore how the localization of these materials – specifically, applying hydrogels directly on the electrode versus incorporating DES and ILs within the electrolyte – affects sensor efficiency and enzyme stability. Hydrogels are typically deposited onto the electrode surface, where their high water content and three-dimensional polymer network create a favorable microenvironment for biomolecule immobilization. This direct contact enhances enzyme stability by maintaining a biocompatible, hydrated interface that mimics natural biological conditions. However, despite their excellent ability to preserve enzyme activity, the low ionic conductivity inherent to hydrogels can impede efficient charge transfer, which is critical for generating a strong electrochemical signal. In contrast, DES and ILs are often integrated into the electrolyte rather than being fixed onto the electrode. Their tunable ionic conductivity facilitates improved electron and ion transport throughout the sensor, leading to faster response times and heightened sensitivity. Nonetheless, when enzymes are dispersed within the electrode, the challenge shifts to maintaining their structural integrity and long-term activity, as the absence of a supportive immobilization matrix may render them more vulnerable to denaturation.

A promising approach to overcome these limitations is the development of hybrid systems that combine the benefits of both configurations.^163–171^ For instance, polymeric DES-based hydrogels (PODES) have demonstrated enhanced enzyme stabilization and analyte diffusion, as seen in the lab-on-a-bead biosensing platform, where alginate-based polymeric DES were successfully used in colorimetric detection.^165^ Similarly, therapeutic DES-assisted hydrogels have been developed for drug delivery, as demonstrated in the encapsulation of curcumin in alginate-chitosan matrices, which improve biomolecule stability and controlled release.^166^

In addition to DES-based hybrid materials, IL-functionalized hydrogels have also been explored for bioelectronics applications. IL-assisted cellulose coating on chitosan hydrogel beads have shown promise as drug carries, offering controlled release and stability.^167^ Moreover, Ureido-Ionic Liquid Mediated Conductive Hydrogels (ULAS) have demonstrated superior mechanical adaptability, enhance electron transfer, and microbial resistance.^171^ Figure 6a illustrates the ULAS hydrogel synthesis and its key electrochemical properties. One of the key features of this hydrogel is its multifunctionality, including self-healing, self-adhesion, and water retention properties (Figure 6b) Notably, its ionic conductivity was increased by a factor of 14.4, compared to conventional hydrogel (Figure 6c), making it highly suitable for bioelectronic devices. Furthermore, this material demonstrates a highly responsive and reversible electrical signal under mechanical deformation, as shown in its strain-resistance curve. Even after 3,000 stretching cycles, the hydrogel maintained its performance (Figure 6d), highlighting its stability for long-term applications in flexible systems.

**Figure 6:**
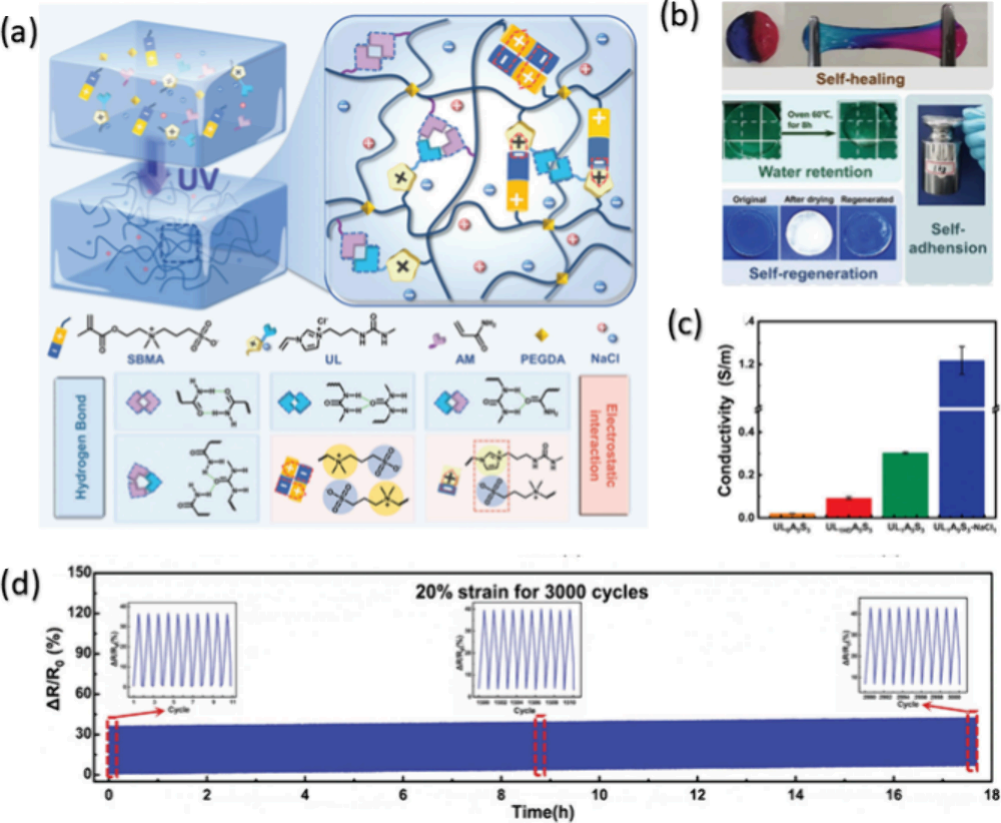
(a) Schematic representation of the ULAS hydrogel structure, highlighting the interactions between SBMA, PEGDA, and NaCl through hydrogen bonding and electrostatic interactions; (b) demonstration of the hydrogel’s self-healing, water retention, self-regeneration, and self-adhesion properties; (c) ionic conductivity of different hydrogel formulations, showing a significant enhancement upon the addition of NaCl; (d) mechanical stability of the hydrogel under 3000 stretching cycles at 20% strain, demonstrating its durability for flexible sensor applications. Reprinted with permission under a CreativeCommons CC BY License from Rui Wang et al., *Advanced Science*, 11, 2307981 (2024). Copyright 2024 John Wiley and Sons.

In conclusion, the choice between hydrogels, DES, and ILs in enzyme-based bioelectronics depends largely on the specific requirements of the application. Each material offers unique advantages in terms of enzyme stabilization, biocompatibility, and performance, though each also presents certain challenges that must be addressed. As research in this field continues, innovations in material science and biosensor design will likely lead to new approaches that combine the best qualities of these materials, resulting in more efficient, reliable, and versatile biosensors for a broad array of applications.

## 6 Challenges and Opportunities

Figure 7 illustrates the complementary roles of hydrogels, DES, and ILs in enzyme stabilization, highlighting their individual advantages, challenges, and the intersections where their properties converge to enhance biosensor performance. Hydrogels, while highly valued for their biocompatibility and effective enzyme immobilization, face a significant challenge in optimizing ionic conductivity. The hydrophilic and porous nature of hydrogels makes them ideal for maintaining enzyme activity in aqueous environments, but this same property limits their ability to conduct ions effectively, which is a critical factor in electrochemical bioelectronics. Since many biosensors rely on the efficient transfer of electrons and ions to generate detectable signals, the limited conductivity of hydrogels can hinder their performance, particularly in electrochemical sensing applications. To overcome this limitation, researchers have explored incorporating conductive materials, such as carbon nanotubes,^172,173^ graphene,^174,175^ or metal nanoparticles,^176,177^ into hydrogel matrices. These materials can enhance the ionic and electronic conductivity without compromising the structural integrity or biocompatibility of the hydrogel. However, finding the right balance between conductivity and biocompatibility remains a challenge. While incorporating conductive additives can improve sensor performance, it may also introduce complexity and potential toxicity, which could reduce the overall safety of hydrogels for in vivo applications. Thus, the opportunity for hydrogels lies in developing hybrid materials that maintain their biocompatibility while significantly enhancing their conductivity, allowing them to be used more broadly in biosensing applications that require high sensitivity and electrochemical precision.

**Figure 7:**
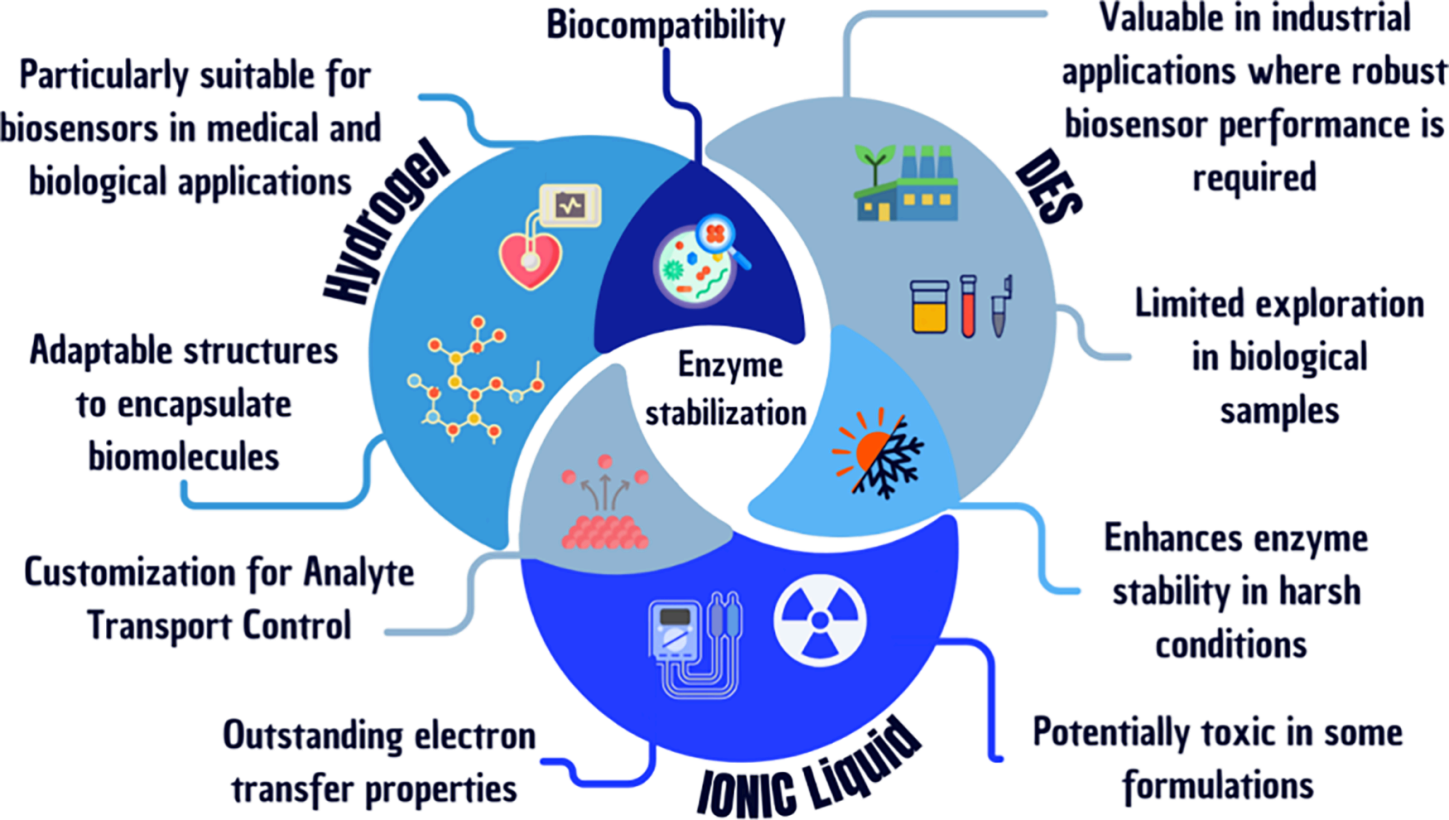
Diagram illustrating the distinct and shared characteristics of hydrogels, deep eutectic solvents (DES), and ionic liquids (ILs) in enzyme-based biosensors.

DES have shown great promise due to their eco-friendly properties, excellent enzyme stabilization capabilities, and adaptability in harsh conditions. However, one of the primary challenges facing DES is scalability. While DES are typically composed of inexpensive and readily available components, such as choline chloride and urea, producing them at a scale suitable for industrial or widespread commercial applications requires optimization of their formulation and manufacturing processes. Inconsistent ratios of HBDs and HBAs or impurities can lead to variations in the physical and chemical properties of DES, which could affect the reproducibility and reliability of bioelectronics. Moreover, the lack of comprehensive studies on the long-term stability of DES in complex biological environments presents another challenge. While DES have proven effective in stabilizing enzymes under controlled laboratory conditions, their performance in real-world biological systems, such as blood or tissue fluids, remains underexplored.^118^ The opportunity for DES lies in expanding their use in more complex biological systems. By further researching the interactions between DES and biological matrices, and by optimizing their scalability, DES could be applied to a wider range of bioelectronics, particularly in medical diagnostics and environmental monitoring. Their biodegradability and low toxicity make them an attractive alternative to conventional solvents and overcoming these scalability issues could position DES as a leading material in sustainable biosensor technologies.

ILs are renowned for their outstanding electron transfer properties, making them highly effective in electrochemical biosensors. However, their high viscosity poses a significant challenge, particularly in applications that require rapid analyte diffusion. The slow movement of molecules within highly viscous ILs can reduce the response time of device, making them less suitable for real-time or dynamic sensing applications. Another major challenge for ILs is toxicity, as certain cation–anion combinations can be harmful to living organisms or the environment. ILs with long hydrophobic alkyl chains tend to exhibit higher toxicity, primarily due to their enhanced interactions with biological membranes and increased disruption of cellular structures.^178^ Despite these challenges, the tunability of ILs presents a significant opportunity. By altering the cation–anion pair, researchers can modify the physical and chemical properties of ILs, potentially reducing viscosity and minimizing toxicity. The development of biocompatible and low-viscosity ILs would open new avenues for their application in bioelectronics particularly in fields like medical diagnostics and wearable biosensing devices. Furthermore, continued innovation in IL synthesis could lead to new formulations that maintain their high electrochemical performance while being safer for use in biological and environmental systems. Reducing viscosity and addressing toxicity concerns are key to improving the widespread application of ILs in enzyme-based bioelectronics, particularly in large-scale commercial and industrial applications.

## 7 Conclusions

Hydrogels are highly effective for enzyme immobilization due to their high-water content and biocompatibility, creating a hydrated environment that preserves enzyme activity while allowing analytes to diffuse through the matrix. This makes them particularly suitable for biomimetic devices in medical and biological applications where direct interaction with tissues or fluids is necessary. However, their limited ionic conductivity and sensitivity to environmental changes, such as pH and temperature fluctuations, restrict their use in electrochemical biosensors that rely on efficient electron transfer. Despite this, hydrogels remain an excellent choice for applications where biocompatibility and long-term stability are priorities, especially in wearable or implantable bioelectronics.

DES stand out for their ability to stabilize enzymes in harsh environments, such as high temperatures or in the presence of organic solvents, making them valuable in industrial applications where robust bioelectronics performance is required. The ionic nature of DES enhances electrochemical properties, enabling better electron transfer and improved sensor sensitivity. Additionally, their biodegradable and eco-friendly composition aligns well with the growing demand for sustainable technologies. However, DES are still underutilized in complex biological systems, and scalability challenges remain a barrier to their broader application. With further research, DES hold the potential to be widely adopted in environmental, food safety, and medical diagnostics.

ILs offer superior electron transfer capabilities and long-term stability, making them a natural fit for electrochemical bioeletronics that require fast response times and high sensitivity. The tunability of ILs allows for the adjustment of their viscosity, conductivity, and hydrophobicity to suit specific applications. However, high viscosity in some ILs can slow down analyte diffusion, and the potential toxicity of certain formulations limits their use in biomedical or environmentally sensitive applications. Innovations in IL synthesis could lead to safer, more versatile ILs that maintain their excellent electrochemical properties while minimizing these drawbacks.

Looking ahead, hydrogels, DES, and ILs will continue to shape the future of bioelectronic technology as research addresses their current limitations and explores new avenues for their application. For hydrogels, the focus will be on enhancing ionic conductivity without sacrificing their biocompatibility. This could involve the development of hybrid materials that incorporate conductive nanoparticles, carbon-based materials or even the integration between hydrogel, DES and ILs. For DES, overcoming scalability issues and expanding their use in biological systems will be crucial for their wider adoption. With their strong performance in enzyme stabilization and their eco-friendly nature, DES are poised to become a key material in sustainable biosensing technologies. Research into how DES interact with complex biological matrices, such as blood or tissue, will help unlock their full potential in medical diagnostics, environmental monitoring, and industrial biosensing applications. ILs are expected to see advances in reducing viscosity and improving biocompatibility, which will allow for their broader use in enzyme-based bioelectronics, particularly in medical and wearable applications. The continued development of new IL formulations that maintain high electron transfer rates while being safer and more adaptable for biological use will open new opportunities in real-time monitoring, point-of-care diagnostics, and industrial sensor systems. By addressing concerns about toxicity and optimizing their performance in large-scale applications, ILs could lead to more efficient and precise biosensing technologies across a wide range of fields.

In summary, hydrogels, DES, and ILs each offer unique advantages that can enhance the performance of enzyme-based biodevices, and continued research will enable their broader use in future technologies. As these materials evolve, they will provide new opportunities for creating more reliable, sensitive, and sustainable devices, expanding the possibilities for real-time diagnostics, environmental monitoring, electrosynthesis, and advanced industrial applications.
